# Building bridges: the integral presence of audiologists and speech-language pathologists in comprehensive interprofessional primary care teams

**DOI:** 10.1017/S1463423626101078

**Published:** 2026-04-10

**Authors:** Grecia Alaniz, Jana Bataineh, Robin O’Hagan, Sheila Moodie, Margaret Pichora-Fuller, Jennifer Cameron-Turley, Bonnie Cooke, Anne Carey, JB Orange, Miranda Cooper, Danielle Glista

**Affiliations:** 1 Health and Rehabilitation Sciences, Western University, London, Canada; 2 Wilson Centre for Research in Education, University Health Network, Toronto, Canada; 3 Department of Physical Therapy, University of Toronto, Toronto, Canada; 4 National Centre for Audiology, Western University, London, Canada; 5 School of Communication Sciences and Disorders, Western University, London, Canada; 6 Department of Psychology, University of Toronto, Toronto, Canada; 7 Department of Gerontology, Simon Fraser University, Burnaby, Canada; 8 Speech and Audiology Canada, Canada; 9 Soins continus Bruyère: Bruyere, Canada; 10 Canadian Centre for Activity and Aging, Western University, London, Canada; 11 Independent Scholar

**Keywords:** audiology, communication disorders, comprehensive primary care, interprofessional team, speech-language pathology

## Abstract

**Aim:**

This article provides an overview of the professional roles of audiologists and speech-language pathologists (S-LPs) in interprofessional primary care.

**Background:**

Current published literature considering primary care delivery within comprehensive interprofessional teams contains little representation of professionals from the fields of audiology and speech-language pathology.

**Methods:**

An illustrative case scenario highlights the key roles of audiologists and S-LPs in primary care, and how collaborative relationships within an interprofessional primary care team structure can enhance the overall quality of care provided to patients and to their families.

**Findings:**

As experts in the prevention, diagnosis, and rehabilitation of communication disorders, with S-LPs supporting speech and swallowing disorders and audiologists supporting hearing and vestibular disorders, S-LPs and audiologists are well-positioned to support meaningful participation in primary care across the lifespan and in collaboration with different healthcare professionals, including patients experiencing cognitive decline.

## Laying down the foundation: the current context of communication health in primary care

Globally, primary care systems are under continuous strain to address the increasingly diverse and complex health needs of patient populations amidst staffing shortages, financial constraints, and burnout (Agarwal *et al*., 2020; Hong *et al*., 2023). In Canada, the focus of care provision is shifting towards primary prevention methods, including promoting healthy aging, in an effort to lessen the burden on the healthcare system (Visconti and Neiterman, 2021). Communication disorders, including conditions that disrupt the complex processes of hearing, language, speech, and swallowing at any point in the lifespan, present regularly in primary care and pose significant barriers to care provision (Devadiga, 2014). As a result, patients are often left with inadequate knowledge or understanding of their health condition or treatment plan. Such barriers are heightened by the limited education and training available to healthcare professionals around speech, language, and hearing-related conditions (Carragher *et al*., 2021; Hur and Kang, 2022). Additionally, heavy clinical workloads and burnout often result in communication disorders being overlooked (Zota *et al*., 2023). Gaps in health professionals’ training for communication disorders reduces access to essential education for patients with communication disorders and their caregivers. Furthermore, inadequate management of these disorders can exacerbate health inequities, impacting patients’ employment and psychosocial well-being (Morris, 2022).

The prevalence and implications of communication disorders on healthcare systems are far-reaching (Pinborough-Zimmerman *et al*., 2007; Devadiga, 2014; Committee on the Evaluation of the Supplemental Security Income (SSI) Disability Program for Children with Speech Disorders and Language Disorders *et al*., 2016; Palmer *et al*., 2019; World Health Organization, 2021; Cummings, 2023). For example, developmental communication milestones for children are critical to identifying complex and chronic conditions, including autism, learning disabilities, literacy difficulties, and attention disorders (Pinborough-Zimmerman *et al*., 2007; Committee on the Evaluation of the Supplemental Security Income (SSI) Disability Program for Children with Speech Disorders and Language Disorders *et al*., 2016; Cummings, 2023). Communication difficulties are highly prevalent, presenting in approximately 10% of school-aged children, indicating a significant number of children with potential complex, chronic conditions (Pinborough-Zimmerman *et al*., 2007; Committee on the Evaluation of the SSI Disability Program for Children with Speech Disorders and Language Disorders *et al*., 2016; Cummings, 2023). Among older adults, hearing loss is the third most common cause of disability, globally, affecting 60% of adults over the age of 60 and is one of a growing list of potentially modifiable risk factors for dementia (World Health Organization [WHO], 2021; Cummings, 2023). Similarly, swallowing disorders, affecting up to 33% of older adults, can lead to serious health consequences such as pulmonary infection, malnutrition, dehydration, poor wound healing, reduced oral medication tolerance, and prolonged hospitalization (Attrill *et al*., 2018; Thiyagalingam *et al*., 2021). Thus, early identification and intervention for communication disorders is instrumental in mitigating their associated lifelong consequences (Giddan and Milling, 1999; Moeller *et al*., 2024).

In Canada, audiologists and S-LPs are autonomous, regulated health professionals with expertise in the prevention, assessment, and management of communication disorders (Speech-Language & Audiology Canada [SAC], 2025). Consequently, S‑LPs and audiologists can independently determine the type, intensity, and frequency of interventions that fall within their scope of practice and are aligned with patients’ needs and goals (SAC, 2025). Depending on provincial coverage rules or private insurance requirements, patients may also self-refer to these services without a physician referral (SAC, 2025). However, both professions have experienced limited integration into the Canadian primary care system, highlighting a practice gap in team-based primary care (Bataineh *et al*., 2024). Recently, efforts have been made within the Canadian healthcare context to improve primary care through the integration of comprehensive interprofessional primary care (CIPC) teams (‘Team Primary Care,’ 2023). These CIPC teams strive to leverage the expertise and knowledge of an interprofessional team to implement the Starfield Principles of Primary Care, originally articulated by Barbara Starfield in 1992 and further developed in 1998, that continue to be relevant today (Starfield, 1992, 1998; Starfield *et al*., 2005). The four principles focus on: (i) *First contact and accessibility*, ensuring primary care serves as the main entry point to the health system; (ii) *Continuity*, fostering ongoing therapeutic relationships between patients and healthcare providers; (iii) *Comprehensiveness*, providing a broad range of services to meet most health needs; and (iv) *Coordination*, which is integral to improving effectiveness, safety, and efficiency of care (Starfield, 1992; Foo *et al*., 2021). This paper aims to demonstrate how the integration of audiologists and S-LPs within CIPC teams offers a viable avenue towards accessible and early preventive care for individuals living with communication, swallowing, and vestibular disorders, while also reducing the growing workloads of an overwhelmed primary care system (Dennis *et al*., 2021).

## Illustrating audiologists’ and S-LPs’ expertise through Daniel’s primary care journey

**Figure 1.**
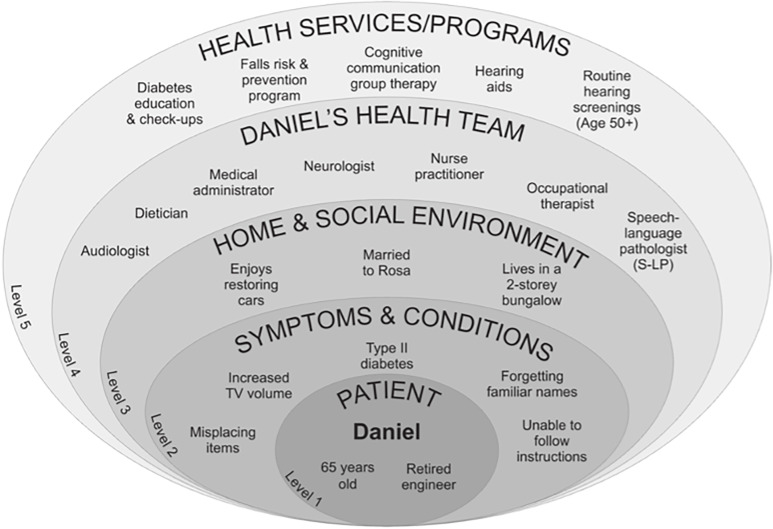
A summary of Daniel’s medical neighbourhood. This multi-level healthcare representation encompasses factors related to the patient (level 1), symptoms & conditions (level 2), home & social environment (level 3), health team (level 4), and service/programme (level 5).

Drawing on the authors’ collective clinical experience, we developed a hypothetical, clinically grounded scenario to illustrate potential roles for audiologists and S‑LPs within a CIPC team within a Canadian primary care context. This scenario will demonstrate how professional expertise can enhance patient-centred care, optimize patient health outcomes, and foster collaborative relationships within the team, in both co-located and separate clinical settings. The inclusion of separate clinical settings is based on the ways Canadian primary care teams are leveraging virtual communication modalities (e.g., telehealth, video-based appointments) and interprofessional care models to meet the needs of patients in their communities, particularly in remote and rural regions of Canada (Young & Young, 2016; Lukey *et al*., 2021; Cronin *et al*., 2025). The use of virtual communication modalities within this scenario is illustrated through the use of a shared electronic medical record (EMR) as a fundamental tool designed to facilitate information-sharing and collaborative care provision within an interprofessional team context. The scenario aims to foster a better understanding of key roles that audiologists and S-LPs can play within CIPC as part of a multi-level healthcare system. In Canadian primary care, this multi-level system is known as a *medical neighbourhood*, encompassing the people, settings, and supports that shape care beyond individual clinical encounters (College of Family Physicians of Canada, 2020). This includes patient characteristics and health concerns, home and social contexts, the interdisciplinary care team, and the services and programmes that coordinate and deliver care over time (College of Family Physicians of Canada, 2020). In this scenario, Daniel is presented as a hypothetical older adult patient whose care is conceptualized within a medical neighbourhood framework. Figure 1 represents Daniel’s medical neighbourhood as a five-level model encompassing patient factors (level 1), symptoms and conditions (level 2), the home and social environment (level 3), the health care team (level 4), and relevant services and programmes (level 5).

### Daniel’s primary care experience

Daniel is a 65-year-old, retired engineer, who attends his annual diabetes check-up with his nurse practitioner (NP). The NP notices Daniel struggling to understand her at times and repeating himself or searching for words during their conversation. The NP asks him if he is experiencing problems with his memory, where Daniel reveals that he sometimes has difficulty remembering his day-to-day whereabouts and activities, prompting her to conduct a mental status screening and make a general neurology referral, with a 6-month wait (Creavin *et al*., 2016). In the meantime, she suggests Daniel meet with the team’s S-LP who has expertise in conducting cognitive-communication tests for early detection of cognition and memory problems, to which Daniel agrees. The NP also notices Daniel has no record of a hearing test. She informs Daniel about the current recommendations for hearing screening (see Figure 1, level 5), which should be conducted regularly starting at 50 years of age, as hearing impairments can contribute to poor cognitive assessment performance (Littlejohn *et al*., 2022). The NP uploads her report to the shared EMR for the team to access (level 4). The medical administrator schedules an appointment with the team’s S-LP and affiliated audiologist through the team’s shared EMR, sends the referral to neurology, and gives Daniel a written reminder of his hearing test for the following week.

During his appointment at the local audiology clinic, the audiologist conducts a comprehensive hearing assessment, finding that Daniel has permanent, moderate binaural hearing loss and is a candidate for bilateral hearing aids. The audiologist discusses Daniel’s results, hearing device options, and at-home communication strategies for Daniel and Rosa, his wife (level 3). Daniel selects a hearing aid model and accessories that fit his lifestyle and the audiologist schedules appointments for the hearing aid fitting and review. During the appointment, the audiologist observes Daniel having difficulty rising from the chair. Given Daniel’s symptoms (level 2), the audiologist conducts a mobility screen and determines that Daniel would benefit from a falls risk assessment and programme (level 5), with Daniel’s consent. This information is recorded in the EMR report.

Prior to Daniel’s speech and language assessment, the S-LP reviews the team’s EMR notes to determine the best standardized tests to identify Daniel’s challenges with communication and cognition, including following commands, verbal repetition, and description of objects. The S-LP interviews Daniel and Rosa to determine their leisure activities, physical activity habits, social connections, sleep patterns, and any other communication-related concerns that they might have. The findings indicate that Daniel is experiencing difficulties with attention, short-term memory, and information processing, consistent with a mild cognitive impairment (MCI; level 2). The S-LP shares with the team via the EMR her concerns regarding the possible presence of MCI. The S-LP, Daniel, and Rosa co-create a plan to leverage Daniel’s cognitive-communication strengths, address his challenges, and support his participation in his preferred activities (level 3). Daniel is scheduled for bi-weekly cognitive-communicative group therapy (level 5), offered through the team.

Within days of wearing his hearing aids, Rosa notices an improvement in Daniel’s attention; however, his memory and word-finding difficulties persist. She also notices that Daniel appears slightly steadier on his feet. At his follow-up appointment, the audiologist confirms that the hearing aids have improved Daniel’s speech-understanding abilities and balance, and educates Daniel and Rosa on the relationships among hearing, balance, diabetes, and cognitive health (Baydan-Aran *et al*., 2023; Koh *et al*., 2015; Samocha-Bonet *et al*., 2021; Sarant *et al*., 2024). The audiologist also reviews effective communication strategies and basic hearing aid care with Daniel and Rosa. She recommends Daniel attend annual check-ups and obtain further testing if he is experiencing changes to his hearing or balance symptoms. Additionally, the audiologist sends a recommendation through the shared EMR used by Daniel’s team for the team to attend an educational session on communication strategies and devices, led by the audiologist and S-LP. This session aims to equip team members with communication techniques and strategies that benefit Daniel and other patients experiencing communication difficulties and enhance their care experience (Glista *et al*., 2024a).

Following the team’s educational session, the EMR sends a prompt to the NP to review the team members’ notes and Daniel’s recent bloodwork. The nurse practitioner requests the medical administrator to call and book an appointment for Daniel and Rosa to review his progress in his therapies and to discuss his bloodwork results. In preparation, the nurse practitioner reviews the EMR, noting each team members’ recommendations regarding Daniel’s hearing, cognition, and mobility.

During the appointment, the NP discusses the team’s recommendations and progress. She emphasizes the importance of utilizing hearing assistive devices to improve communication ability, and in maintaining a routine around physical activity, cognitive-communication therapy, and a balanced diet as key strategies to mitigate some of the risk factors associated with MCI that could, in turn, progress to dementia. The nurse practitioner recommends Daniel meet with the team’s dietitian (level 4) to review his diet, as his bloodwork indicates elevated blood glucose levels. She also suggests seeing the occupational therapist (OT; level 4) for the falls risk prevention programme (level 5). Daniel agrees to book both appointments.

Prior to Daniel’s appointments, the EMR prompts the OT and dietitian to review communication recommendations (e.g., hearing aid check) from the S-LP and audiologist prior to Daniel’s appointments. Using the recommended strategies, the OT assesses Daniel’s balance and determines he has a mild falls risk. The OT recommends a 6-week group balance and strengthening programme to minimize his risk of falls. The dietitian reviews Daniel and Rosa’s current diet, meal preparation, and cooking routines and provides education and strategies to improve Daniel’s blood glucose levels. Together, they create a feasible plan that works for both Daniel and Rosa since they both cook in their household. They also decide on monthly follow-up appointments to review their progress.

Next, Daniel and Rosa attend his neurology appointment. Based on team members’ reports, Daniel’s laboratory results, and the neurologist’s clinical examination, the neurologist confirms that Daniel does show MCI and ongoing monitoring for dementia will be needed. Neuroimaging is recommended and a requisition is issued for an MRI. In the meantime, the neurologist prescribes Daniel medication to improve his memory and judgement and advises him to continue with the team’s recommendations as much as possible. The neurologist’s office schedules a 6-month follow-up appointment and enters the report into the EMR.

Throughout the next 6 months, Daniel attends his follow-up appointments with his primary care team and follows their recommendations. Daniel and Rosa recognize that attending to hearing, physical activity, diet, social connections, communication, and/or cognitive health can potentially modify the trajectory of Daniel’s MCI diagnosis (Xiao *et al*., 2006; Hughes *et al*., 2013; McGrattan *et al*., 2018; Bucholc *et al*., 2022; Zhu *et al*., 2023). The team also recognizes that many providers are involved in Daniel’s care and work closely with Daniel and Rosa to ensure the frequency of appointments is feasible. If, and when, Daniel and Rosa feel there are too many appointments, the team will work to accommodate them by booking appointments back-to-back when possible, offering virtual care options, and providing home visits when appropriate.

### The role of audiologists

Daniel’s case highlights two important aspects of the roles and expertise of audiologists in primary care including their abilities to identify and to treat hearing loss, and to triage common risk factors for hearing loss and balance (i.e., diabetes; Pichora-Fuller *et al*., 2015). Research has demonstrated that cognitive test accuracy can be limited when mild hearing impairment is present, due to the standard verbal administration of these tests (Dupuis *et al*., 2015; Kolberg *et al*., 2024). For Daniel, it was necessary to address his hearing loss before proceeding with additional neurocognitive testing to ensure accurate screening. Additionally, hearing loss has been associated with reduced static balance, increasing the risk of falls (Jiam *et al*., 2016; Negahban *et al*., 2017). Audiologists’ training in vestibular and balance disorders provide the opportunity to identify factors that contribute to balance and falls risk, benefitting musculoskeletal intervention, and assists in a comprehensive care process, in collaboration with other health care team professionals, such as the OT in Daniel’s case. For older adult patients, audiologists can provide intervention for hearing, vestibular, and balance disorders, including counselling, treatment, management, and rehabilitation, integral components in cognitive care and falls prevention (SAC, 2016).

### The role of S-LPs

Daniel’s case illustrates S-LPs’ expertise in conducting cognitive-communication screenings, assessments, and implementing therapeutic interventions. Research shows that cognitive-communication therapies can significantly improve memory function and prevent further decline (Bosco *et al*., 2018). Given that older adult patients can experience a worsening of symptoms while awaiting specialist appointments, S-LPs provide essential, timely care that supports ongoing management of communication needs, including when access to specialist services is delayed. Additionally, swallowing difficulties can emerge with the progression of MCI to specific types of dementia, where the management of dysphagia falls directly within the scope of practice of S-LPs, further emphasizing their important role in managing a broad range of symptoms associated with cognitive decline (Pichora-Fuller *et al*., 2015).

## Bridging opportunities for audiologists and S-LPs in CIPC

As demonstrated in the case scenario, the integration of audiologists and S-LPs within a CIPC team enhances the quality of primary care provision by enabling a comprehensive understanding of patient needs, particularly for patients experiencing chronic, complex conditions, including communication disorders. Moreover, the timely and efficient exchange of information via the EMR optimizes care, enhances timely care management, and reduces health care costs. In this section, we outline key considerations for integrating audiologists and S-LPs into CIPC, including (1) the ability to act as a first point of contact, (2) the importance of delegating services, (3) using EMR systems to enhance interprofessional communication and collaboration, and (4) trusting team members’ knowledge and expertise.

### First point of contact

Within team-based primary care, family physicians (also known as general practitioners outside of Canada) and NPs can operationalize Starfield’s first contact/accessibility principle by enabling audiologists and S‑LPs to serve as *first-contact providers* for concerns within their regulated scopes of practice. This approach, which has been implemented for other professions (e.g., physiotherapists and midwives) in select Canadian primary care teams, can improve access by reducing unnecessary steps to care and by ensuring patients reach the most appropriate provider earlier (Vader *et al*., 2022; Darling *et al*., 2025). Shifting initial screening for hearing and communication-related concerns directly to audiologists and S‑LPs means CIPC teams can optimize each team member’s expertise, reduce unnecessary demand on physicians and NPs, and streamline care pathways so patients receive timely, appropriate support, while reserving physician/NP visits for needs requiring medical assessment or broader diagnostic workup (Kiran, 2022; Glazier, 2023). In the scenario, Daniel required an initial NP visit to determine his appropriate care pathways. However, in future, he could directly access these providers for new or recurring hearing and communication-related concerns, as long as his primary care team enables audiologists and S‑LPs as first-contact providers.

### Service delegation

The current climate of primary care has required innovative ways to engage in care provision. Service delegation improves access to care by leveraging health providers overlapping skills and expertise (CIHC, 2024; Brown *et al*., 2021). This delegation allows various providers to deliver care, rather than depending on just one provider, and enables patients to access care from a range of available and appropriate providers. However, successful service delegation relies upon role clarification and negotiation, both of which are key competencies in the CIHC Interprofessional Competency Framework (CIHC, 2024). *Role clarification* refers to a thorough understanding of each team member’s role and scopes of practice, whereas *role negotiation* refers to a collaborative decision-making process that engages providers in roles that benefit the team *and* patient. In Daniel’s case, the audiologist identified and screened his balance symptoms, and the S-LP provided Daniel cognitive-communicative therapy, which can overlap with the OT’s scope of practice. However, given Daniel’s symptoms and his OT’s clinical focus on falls prevention, the audiologist and S-LP were the most suitable professionals to attend to him, given their expertise in communication disorders. This allowed Daniel to receive ongoing care tailored to his cognitive-communication needs. Thus, diverse primary care teams that adopt collaborative, interprofessional care approaches can provide high-quality care, while relieving the strain on the primary care system, particularly in the current climate of rapidly increasing physician burnout (Dennis *et al*., 2021).

### EMR as a collaborative communication tool

Healthcare systems globally are seeing increased technological advancements, such as EMR systems (O’Malley *et al*., 2015; Kosteniuk *et al*., 2024). EMR systems can effectively serve as information sharing platforms, facilitating service delegation as well as enhancing communication and collaboration within interprofessional primary care teams (O’Malley *et al*., 2015; Kosteniuk *et al*., 2024). More specifically, the information sharing capacities of EMR systems allow for easier access to other providers’ findings and recommendations, facilitating cohesive, integrated care provision for patients (Kosteniuk *et al*., 2024). These benefits of EMR systems apply whether teams are co-located or working remotely (O’Malley *et al*., 2015; Kosteniuk *et al*., 2024). This flexibility is increasingly important in healthcare, where providing remote care and maintaining seamless communication between healthcare professionals is crucial (O’Malley *et al*., 2015; Kosteniuk *et al*., 2024). Furthermore, we highlight this well-documented utility of EMR to emphasize that while co-location is advantageous, it is not necessary to encourage collaborative relationships. Collaborative relationships can be fostered with existing community providers through collaborative communication tools, such as EMR, as illustrated by the audiologist in Daniel’s case. Lastly, although not illustrated in the scenario, EMR systems are increasingly incorporating patient-facing communication functionalities (e.g., secure messaging, appointment reminders, and access to care plans and results), aligning with primary care goals related to timely access and comprehensiveness (Gorfinkel and Lexchin, 2018; Tapuria *et al*., 2021).

### Trust in team expertise

The transition from a siloed health care delivery model to an integrated primary care model can pose challenges to collaborative efforts. The introduction of new professions into an existing care model can lead to issues of trust related to professional service boundaries, and uncertainty regarding the knowledge and competence of these new roles (Brown *et al*., 2021). Within the context of Canadian primary care, audiologists and S-LPs have been overlooked (Bataineh *et al*., 2024). The lack of inclusion of audiologists and S-LPs in primary care can perpetuate misconceptions regarding their knowledge and expertise among primary care professions, limiting audiologists and S-LPs potential contribution to patient management within a CIPC team. Trust, a crucial component of teamwork, relies on respect and communication across individual, team, and organizational levels (Hamilton *et al*., 2024). In an effort to improve health providers’ trust and understanding of audiologists’ and S-LPs’ roles within CIPC, educational modules have been developed by the authors of this paper (Glista *et al*., 2024b; Moodie *et al*., 2024). These modules focus on the roles and scopes of practice for audiologists and S-LPs, and how they can support the goals of primary care. Such educational tools can pave the way for the provision of collaborative care, and the enhancement of care for patients with complex communication disorders. As primary care responds to current pressures, it becomes crucial for organizations, teams, and providers to willingly collaborate with new team members to fully leverage the advantages of CIPC teams.

## Closing the gap: integrating audiologists and speech-language pathologists in CIPC

The need for comprehensive, interprofessional approaches to primary care, to alleviate the ongoing strains on primary care systems, is well-documented (Gougeon *et al*., 2017; Smeets *et al*., 2020; Ohta *et al*., 2021). Our illustrative, hypothetical case highlights the importance of integrating diverse scopes and skills within patient-centred CIPC, consistent with the WHO’s *Integrated Care for Older People* (ICOPE) framework, which promotes primary care pathways for dementia and hearing care as best practice (WHO, 2025). Additionally, we discussed key considerations for successful integration of audiologists and S-LPs into CIPC teams. Specifically, we highlighted the need for role clarification and negotiation as prerequisites for service delegation, the utility of EMR systems as a tool that fosters interprofessional communication and collaboration, and the importance of trust in providers’ knowledge and expertise at the organizational, team, and individual levels. It is important to acknowledge that addressing these gaps in primary care will necessitate changes in both education and practice. This involves shaping clinical practice and educational efforts within health professions training and among practising health professionals to adopt comprehensive and team-based approaches to primary care. As primary care teams look for innovative ways to expand and engage with CIPC, we hope to see audiologists and S-LPs become more prominent across these teams.
